# Supplementary material for: Synergizing habits and goals with variational Bayes
Source: Nat Commun. 2024 May 25;15:4461. doi: 10.1038/s41467-024-48577-7 (PMC11525633; doi:10.1038/s41467-024-48577-7)
Supplement: Supplementary file 1 — Supplementary Information [file 41467_2024_48577_MOESM1_ESM.pdf]

# Supplementary Information for “Synergizing habits and goals with variational Bayes”

## SUPPLEMENTARY RESULTS

### Balance between complexity and accuracy in free energy

Since the complexity (KL-divergence) term plays a key role in our framework, we empirically investigate the effect of changing the weight ($\beta_z$ in Equation (7)) of the complexity term in free energy[1]. The results are shown in Supplementary Figure 1. We found that large or smaller $\beta_z$ does not significantly affect the performance in terms of mean rewards in each trial (Supplementary Figure 1a,f,k). However, a smaller $\beta_z$ leads to lower uncertainty of the prior intention and thus a higher habitual ratio (Supplementary Figure 1b,c,g,h,l,m). This is due to the weaker regularization by the KL-divergence (Supplementary Figure 1d,i,n) between habitual and goal-directed intentions, such that the habitual system can develop more accurate intention conditioned on current observation, with less constraint to care about the final outcome. In addition, the prediction loss becomes large when $\beta_z$ is very large (Supplementary Figure 1e,j,o), since the complexity constraint is too strong.

### Effect of intention precision threshold

During goal-directed AIf, we have introduced an early-stop mechanism (see Figure 1d and **Methods**) to save computational cost. The AIf process terminates when the synergized intention's variance $(\sigma_t^s)^2 = 1/((\sigma_t^q)^{-2} + (\sigma_t^p)^{-2})$ is small enough, or more specifically when $\sigma_t^s < \sigma^{\text{thres}}$, where $\sigma^{\text{thres}}$ is the precision threshold. To evaluate how the precision threshold affects the agent's behavior, we have conducted simulations of the habitization experiment (Figure 2). The results are shown in Supplementary Figure 2. As expected, the number of iterations needed in AIf increases when the precision threshold $\sigma^{\text{thres}}$ becomes small (Supplementary Figure 2e,k,q), since the goal-directed intention requires more AIf iterations to become more precise. A medium value of $\sigma^{\text{thres}}$ also results in a faster re-adaptation in stage 3 (Supplementary Figure 2m,r). However, increasing or decreasing $\sigma^{\text{thres}}$ in a reasonably wide range (0.01 to 0.2) does not significantly affect other main results (Supplementary Figures 2, 3, 4).

### Learned representation of the latent intention

Our framework reflects one of the core ideas of predictive coding[2] that the internal representation of the network should be more compressed than the raw observation. In our case, the dimension of the intention variable is 4, meaning a condensed representation of the current vision (64 by 16 pixels) and the goal. To take a closer look at how $z_t^q$ and $z_t^p$ encode the information need for behaving, we visualize the intention in various ways (Supplementary Figure 5). We are interested in the representation learned in the adaptation stage of the second experiment (Figure 5a), since the agent has developed diverse behavior to go to the left or right exit randomly (Figure 5c, Supplementary Figure 5a,b). We plot the representation of intention sequence in each trial (Supplementary Figure 5b), and intention at each step (Supplementary Figure 5d,e). It can be seen that the intention may not reflect the final exit at the beginning of each episode, but then both the prior and posterior intentions experience a relatively smooth bifurcation process[3] (Supplementary Figure 5d,e), indicating a progressively strengthening of the certainty about goal-achievability.

## SUPLLEMENTARY PROOF

Here we formally derive the free energy terms in Equation (7). Consider that an agent is learning a model of the world, i.e., trying to predict its current and future observations of the environment; the log-likelihood of correct prediction is being maximized. The log-likelihood is written as $\ln P(x_t, x_{t'} = \bar{x}_t, \bar{x}_{t'})$, where $\bar{x}_t$ and $\bar{x}_{t'}$ are the actual observations at timestep $t$ and $t' > t$, respectively. However, this likelihood is practically intractable, i.e., cannot be written by an analytical function. Therefore, a latent variable $z_t$ is introduced, which follows a tractable distribution, and we train a decoder $P(x_t, x_{t'} | z_t)$ together with the distribution of $z_t$ to approximate the true distribution of $x_t$.

$$\ln P(x_t, x_{t'}) = \ln \int_{z_t} P(x_t, x_{t'}, z_t) dz_t. \tag{1}$$

$$= \ln \int_{z_t} P(x_t, x_{t'} | z_t) p(z_t) dz_t, \tag{2}$$

where $p(z_t)$ is probability density function (PDF) of the prior distribution of $z$, which depends on the current and historical observations $\bar{x}_{1:t}$. Note that here the probability of correct prediction $P(x_t, x_{t'} = \bar{x}_t, \bar{x}_{t'})$ is denoted by $P(x_t, x_{t'})$ for simplicity. A trick is to introduce a variational posterior $z_t^q$ that follows the posterior distribution $q(z_t)$ that depends on future observation $\bar{x}_{t'}$ (where $t' > t$) in addition to $\bar{x}_{1:t}$. We have

$$\ln P(x_t, x_{t'}) = \ln \int_{z_t} P(x_t, x_{t'}|z_t) p(z_t) \frac{q(z_t)}{q(z_t)} dz_t \tag{3}$$

$$= \ln \int_{z_t} \frac{P(x_t, x_{t'}|z_t) p(z_t)}{q(z_t)} q(z_t) dz_t \tag{4}$$

$$= \ln \mathbb{E}_{q(z_t)} \left[ \frac{P(x_t, x_{t'}|z_t) p(z_t)}{q(z_t)} \right] \tag{5}$$

Since the logarithm function is concave, Jensen's inequality implies that

$$\ln P(x_t, x_{t'}) \geq \mathbb{E}_{q(z_t)} \left[ \ln \frac{P(x_t, x_{t'}|z_t) p(z_t)}{q(z_t)} \right] \tag{6}$$

$$= \mathbb{E}_{q(z_t)} \left[ \ln P(x_t, x_{t'}|z_t) \frac{p(z_t)}{q(z_t)} \right] \tag{7}$$

$$= \left\{ \mathbb{E}_{q(z_t)} \left[ \ln P(x_t, x_{t'}|z_t) \right] + \mathbb{E}_{q(z)} \ln \frac{p(z_t)}{q(z_t)} \right\} \tag{8}$$

$$= \left\{ \mathbb{E}_{q(z_t)} \left[ \ln P(x_t, x_{t'}|z_t) \right] - D_{\mathrm{KL}} \left[ q(z_t) \| p(z_t) \right] \right\}. \tag{9}$$

The last step uses the definition of KL-divergence[4]. Equation 9 is known as the variational lower bound or the evidence lower bound (ELBO)[5]. By maximizing ELBO, the original log-likelihood of correct prediction $\ln P(x_t, x_{t'})$ is also increased, thus the agent learns to better predict the environmental observation. The free energy $\mathscr{F}$ is mathematically equal to the negative of ELBO, which should instead be minimized. For each step $t$,

$$\mathscr{F}_t = \underbrace{-\mathbb{E}_{q(z_t|\bar{x}_{1:t}, \bar{x}_{t'})} \left[ \ln P(x_t, x_{t'}|z_t) \right]}_{\text{posterior prediction error}} + \underbrace{D_{\mathrm{KL}} \left[ q(z_t|\bar{x}_{1:t}, \bar{x}_{t'}) \| p(z_t|\bar{x}_{1:t}) \right]}_{\text{complexity}}. \tag{10}$$

In practice, the complexity term is often multiplied by a scalar hyperparameter $\beta_z$ to better balance complexity and accuracy[1]. In our case, the agent predicts both the current and future observations, using $P(x_t|z_t)$ and $P(x_t|z_{t-1})$. Since we use an RNN to encode contextual cues $\bar{x}_{1:t}$ into the RNN state $h_t$ (Figure 1), we finally have

$$\mathscr{F}'_t = \underbrace{-\mathbb{E}_{q(z_t|h_t, \bar{x}_{t'})} \left[ \ln P(x_t, x_{t'}|z_t) \right]}_{\text{posterior prediction error}} + \underbrace{\beta_z D_{\mathrm{KL}} \left[ q(z_t|h_t, \bar{x}_{t'}) \| p(z_t|h_t) \right]}_{\text{complexity}}. \tag{11}$$

Hence, we have obtained the free energy in Equation (7).

# SUPPLEMENTARY DISCUSSION

## Related Work

Some elements in our framework have been discussed in the literature or shared similar ideas. However, our framework is not an incremental study that is merely a straightforward combination of them of them. This section clarifies the novelty of our methodologies (problem definition, optimizing algorithm, and model architecture) given existing ones.

### *Interpretation of habits and goals under active inference theory*

Friston et al.[6] proposed a theoretical perspective on the formation of habitual and goal-directed behaviors under the free energy principle and the active inference (AIf) theory[7]. The paper argued that the key difference between the two behaviors is that goal-directed behavior is belief-based (a latent variable encoding the agent's estimation about current and future states), rather than model-based[8]; while habitual behavior is belief-free rather than model-free: actions are taken without the need to consider or update beliefs about the state of the environment or its future state, and habits can be shaped through repetitively observing the goal-directed behavior. Moreover, a recent theoretical work by Schwöbel et al.[9] proposed a Bayesian perspective for habitual and goal-directed behavior, also under the AIF framework. They consider the likelihood of an agent's behavior as the multiplication of the likelihood of habitual behavior and the likelihood of goal accomplishment, which shares a similar high-level idea with our work. Consistent with these two studies[6,9], our framework also argues that goal-directed behavior is computed by

AIf, and habitual intention, computed from sensory inputs, is trying to approximate the goal-directed intention by minimizing their divergence. However, while these two studies[6,9] question about the necessity of using model-free reinforcement learning (RL) for action learning, tremendous neuroscientific evidence has supported the existence of model-free RL in the brain[8,10–14]. Our study takes a significant step in the computational conceptualization of decision making by incorporating model-free RL with model-based AIf in the same framework, synergizing the power of RL to optimize motor skills and the capacity of AIf to flexibly perform goal-directed planning. Furthermore, our framework does not model the likelihood of motor outputs like in Friston et al.[6] and Schwöbel et al.[9], but rather the latent intention, for habitual and goal-directed behavior. In terms of computer simulations, they assumed categorical (discrete) states and actions, as well as known world models, while our work simulates sensorimotor tasks with visual sensation and continuous motor actions where the agent learns both the world model and motor skills by self-exploration *tabula rasa*, thanks to deep RL[15]. Furthermore, we demonstrate the capacity of our framework to perform flexible goal-directed planning for unseen goals, which were not covered in their experiments[6,9].

### *Deep active inference*

Active inference (AIf)[6,16] has recently been equipped with neural networks (a.k.a. deep AIf) to solve more challenging tasks, including simulated decision problems[17–20] and planning with real robots[21–25].

Deep AIf shares a similar idea with MBRL in terms of inferring actions to achieve desired outcome using an environment model, while the main difference is that AIf maximizes the likelihood to achieve a certain state, while MBRL maximizes expected rewards. Another notable difference is that AIf is a probabilistic framework, while MBRL does not have to be.

Goal-directed planning in our framework employed the idea of active inference. However, our model does not directly infer actions to achieve the goal, but the latent state in the model that encodes the intention. The latent state can also be understood as high-level action from the perspective of hierarchical RL[26,27].

### *Embodied artificial general intelligence*

Let us first look at position papers on how to create a general intelligent embodied artificial agent (also known as the foundation model[28], which refers to AI models that can perform a wide range of tasks and adapt to new challenges). Although no work has yet achieved this goal, there are countless articles that address this problem. Here, we discuss two particularly interesting and related ideas.

The *one big net* framework proposed by Schmidhuber[29] stems from the observation that humans and other animals have one large neural network (i.e. their brain) that can efficiently learn and perform a wide range of tasks. Schmidhuber envisions that such a network would be able to continually learn and adapt to new tasks by reusing and transferring knowledge across tasks, making the learning process more efficient. A shared key idea between our framework and the one big net is that the center part of the model is an RNN. The RNN models the physical dynamics of the world, which is intrinsically invariant with respect to time, and maintains its internal states which can theoretically encode information from an infinitely long history[30]. However, it is not explicitly pointed out how prediction plays a role in goal-directed planning.

Another recent perspective from Lecun is the so-called *autonomous machine intelligence* framework[31], which shares some common high-level ideas with ours. Lecun emphasizes that the world model, which plays a two-fold role of planning for future and estimating missing observations, should be an energy-based model. In the context of goal-directed behavior, the model takes the current state, the goal, and the action to take as input and outputs a scalar energy value to describe their "consistency". Similarly to our ideas, the model and internal states are optimized for energy minimization with gradient methods. A key difference is that our model makes explicit predictions about sensation and that the uncertainty of sensation is handled by the stochastic latent variable z with variational Bayesian methods.

### *Model-based RL*

Model-based RL (MBRL) approaches train the agent(s) based on a mathematical model that can predict the upcoming state or observation given current and previous observations and actions. Usually, the model is used either for dreaming, i.e., generating imaginary experiences to train the agent[32–35] or planning, i.e., inferring the policy that leads to maximum returns in the future[36–38]. In particular, there are also methods that use the model to extract information from the environment to serve model-free RL[39–41], which share a similar idea with our framework for learning habitual behavior. Although several studies[39–41] also used variational RNNs, they focused on single-goal tasks. On the contrary, the stochasticity in our model reaches its potential to enable the agent to randomly pursue one of multiple goals. The planning phase of our framework used active inference[42], which infers the policy using the model as in MBRL, but not to maximize returns but minimize free energy w.r.t. the goal.

### *Variational Bayes in deep learning and RL*

Variational Bayesian (VB) approaches in deep learning have been popular since the introduction of the variational auto-encoder (VAE)[5,43]. The core idea is to maximize the variational lower bound of an objective function of a probabilistic variable so that we can replace the original distribution with a variational approximation[44]. Chung et al.[45] complemented VAE with recurrent

connections by proposing variational RNNs. Variational RNN and its variants were later used in deep RL, e.g., as the world model in MBRL[35,36,40,41] and for approximating unobservable environmental states[39,46]. One critical reason to use VB models in RL is that they are believed to extract useful representation of the environmental state from raw observations[44] and are robust in training. The acquired representation is then incorporated for the original RL task, i.e. maximizing rewards. While our framework can be considered as a new member of the VB family that handles decision-making/control tasks, our idea of modeling habits and goals using variational Bayes has not been discussed in previous deep learning studies.

### *Goal-conditioned RL*

Goal-conditioned RL (GCRL)[47] addresses scenarios in which a goal is given in each episode and needs to be achieved. The goal can be a property or feature[48], observation or state[49], or a language description[50]. The main difference between GCRL and our framework is that in GCRL, goals are given during learning, and agents are only rewarded when the given goal is achieved. In contrast, our framework does not assign any specific goals during training; it is only maximizing rewards for habitual behavior and minimizing free energy expectation. In simple terms, the training and testing problems are consistent in GCRL but different in the proposed framework.

### *Control as probabilistic inference*

Control as probabilistic inference (CPI)[51] proposes using probabilistic inference to compute the optimal control action instead of designing a deterministic control policy, by casting the control task as a probabilistic inference problem over latent variables that describe the state of the system. Although the basic idea shares insights with model-based learning, CPI does not consider detailed outcomes of action, but only maximizes rewards. Therefore, the practical implementation of CPI has turned out to be model-free algorithms, such as soft actor-critic (SAC)[52]. In our implementation, SAC is used as the base RL algorithm to learn the habitual behavior. Readers are also encouraged to refer to related literature[53,54] for in-depth discussion on the relationship between probabilistic inference and decision-making/control.

### *Self-discovery of goals or skills*

A class of methods known as variational skill discovery (VSD)[55] aims to discover action primitives in reinforcement learning (RL) by optimizing an unsupervised or self-supervised objective function based on information theory. These methods use a latent variable, $z$, to label action primitives, which can be discrete[55–57] or continuous[58,59]. The policy model, $\pi(a|z,s)$, where $a$ is action and $s$ is state, is then trained using pseudo (intrinsic) rewards and RL. These pseudo-rewards reflect an information-theoretic objective that encourages the skills to be diverse and predictable by states, such as the variational lower bound of the mutual information between the set of skills and skill termination states.

A particularly related work is from Mendonca et al.[60], which, like our work, considers both control without and with a goal. However, they explicitly train a goal achieving agent to achieve goals using RL by designing a “goal achievement reward” in addition to training an agent for exploration; While in our framework, there is no training for goal achieving.

## Extensive discussion

### *The frame problem of AI*

The frame problem in AI[61] refers to the challenge of determining which aspects of an environment are relevant when making decisions or solving problems. The issue arises due to the vast number of potential factors that an AI system must consider, making it computationally infeasible to model all possibilities. More practically, computational models that take the relevant information as input are vulnerable to the frame problem. Goal-directed behavior is more susceptible to the frame problem, as goals can be highly diverse and complex even with a single sensory modality like vision, not to mention that biological agents possess multiple sensory inputs. Rather than treating the goal as a direct input to the model[49], our framework uses a backward process to infer goal-directed intentions using predictive coding. This approach presents a potential solution to the frame problem in complex environments.

### *Where comes the goal*

While the proposed framework answered the question of how the goal-directed behavior uses habitual skills, a more fundamental question remains: Where does the goal come from? In our simulations and many other machine learning studies[49,60], the goal is assigned by the programmer per task need. But what about in humans and animals? It would be interesting to consider modeling the intrinsic mechanisms of goal selection[62] in future research. A potential mechanism is to learn or evolve a “meta”-habitual behavior of goal-selection that enhance the fitness of the agent and the population.

### *Predictive coding and information bottleneck*

The theory of predictive coding (PC) suggests that the brain learns to identify patterns and reduce unneeded information by removing items from the input that can be predicted based on these patterns in the natural world[2]. In our experiments, information about the environment and the goal is encoded with a low-dimensional vector $z$, while the model can still make

reasonable predictions of future observations, which reflects the key idea of PC. This compact encoding together with the complexity constrain (the KL-divergence between posterior and prior) enables effective active inference to compute the optimal value of goal-directed intention with a constrained, small search space.

Another perspective from machine learning theory is that given the prior of $z$ is unit Gaussian in our cases, minimizing the expected free energy, or the negative variational lower bound[5], can be considered as a special case of the information bottleneck (IB) objective[44,63]. The IB objective tends to minimize the mutual information between the input (vision here) and the latent encoding $z$ and maximize the mutual information between $z$ and the model's prediction[44]. This idea is consistent with PC and provides a mathematical understanding of how minimizing the free energy in training relates to PC.

### *Hierarchy in predictive coding*

Hierarchy is a crucial property in PC because it enables efficient processing of sensory information by allowing the brain to make predictions at different levels of abstraction. For example, neurons in the primary visual cortex have simple and complex receptive fields, while higher-level visual areas have increasingly complex receptive fields that allow for sophisticated processing of visual information, ultimately leading to object recognition[64]. In our case, the RNN state $h$ and intention $z$ can be considered as two levels of task representation, where $z$ is a higher-level abstraction (with only 2 dimensions compared to 256 dimensions of $h$). Nevertheless, future work should consider the intrinsic hierarchy of the model by borrowing ideas from brain science studies, such as the multiple timescale property found in cortical layers[65], or from deep learning models, such as the Swin Transformer[66].

### *Insights for neurological diseases*

Our framework poses a variational Bayesian understanding of habitual and goal-directed behaviors, which may also provide valuable insights to understand and treat neurological diseases such as Parkinson's disease (PD)[67] and autism spectrum disorder (ASD)[68].

For PD, previous research has suggested that patients with PD have difficulty with goal-directed behavior, as they tend to rely more heavily on habitual than goal-directed actions as their goal-directed planning ability is impaired[69,70]. As we have discussed the arbitration between the two types of behavior, this impairment may be explained by a large uncertainty of the goal-directed intention. It might be worth investigating how to reduce the uncertainty of goal-directed intention through medicine / deep brain stimulation (changing internal states)[71,72] or sensory stimulus (changing brain inputs)[73,74] for improving the motor control ability of PD patients.

It is well known that repetitive behavior is a key characteristic of ASD and abnormal predictive coding in people with ASD is a popular explanation[68,75–77]. In particular, the pathology of ASD can be computationally explained by that overweighting the complexity term in free energy impairs cognitive-behavioral flexibility when adapting to a changed environment[78]. Our framework can be used as a computational tool to help understand how the stochasticity of $z$ affects behavioral diversity.

### *Goals and prompts in large language models*

Talking about flexible goals, our framework has a similar idea to the pre-training paradigm of recent advances in AI such as GPT[79] and CLIP[80]. In particular, designing a learning objective function for a given goal is like designing a *prompt* (for language models, a *prompt* is a piece of text that serves as input to generate a continuation or completion, often used to guide its output). However, a key feature of our framework is that its training process does not need to involve goals, which is different from the training of GPT where prompts are used in training. The flexibility of goal-directed planning in our framework originates from the fact that the model only predicts subsequent observation, and any goal in the future can be decomposed into many consecutive steps conditioned on the intention. Importantly, bounding the goal-directed intention's discrepancy from the prior distribution compresses the search space to make the search plausible.

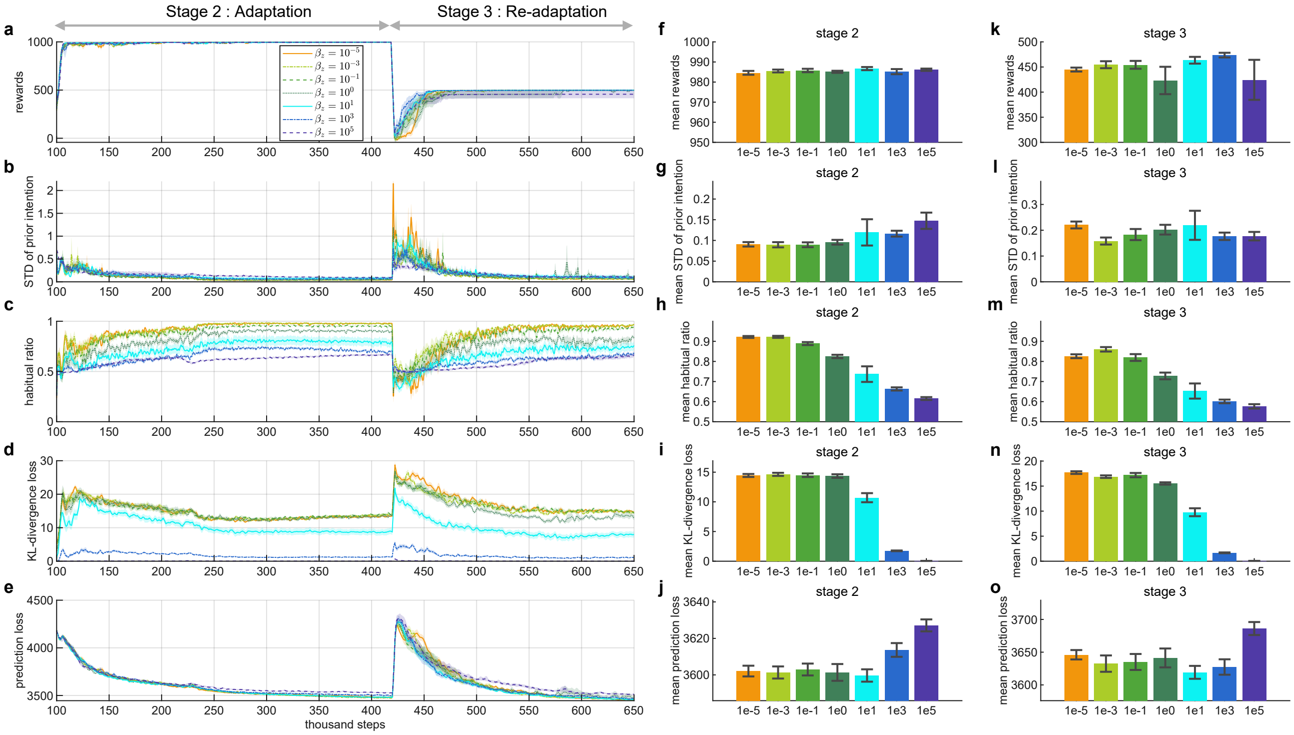


**Supplementary Figure 1.** **Sweeping the weight of the complexity term in free energy** (i.e. $\beta_z$ in Equation (7) for the habitization experiment (Figure 2a). **a-e** Profile of episodic rewards, prior intention STD, habitual ratio, KL-divergence and prediction loss, respectively. **f-j** Their mean values in stage 2. **k-o** Their mean values in stage 3. The results are from 12 random seeds for each case.

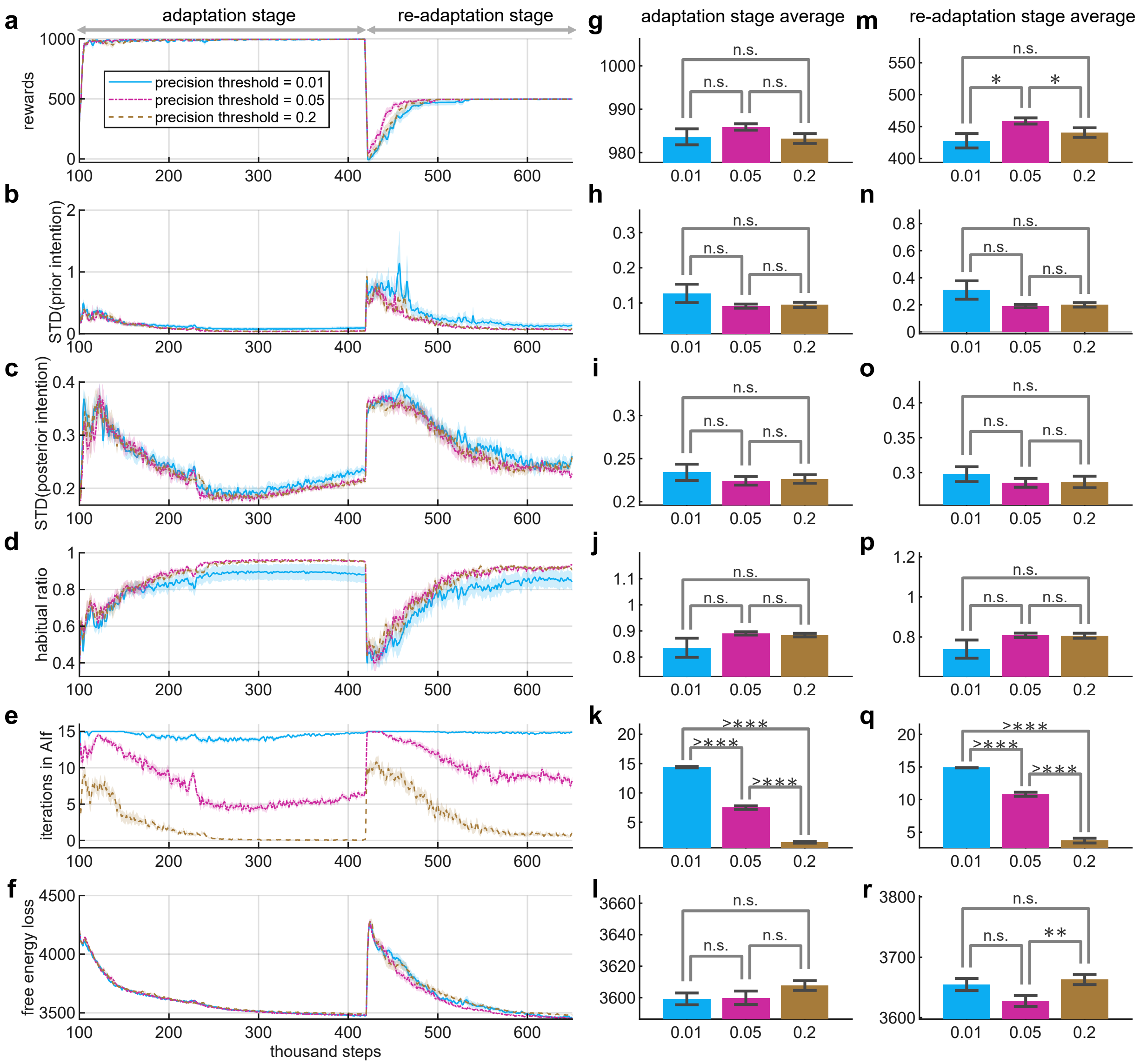


**Supplementary Figure 2. Sweeping the precision threshold for early-stopping active inference** (i.e. $\sigma^{\mathrm{thres}}$ for the habitization experiment (Figure 2a). The results in the main texts were all obtained with $\sigma^{\mathrm{thres}} = 0.05$. **a-f** Profile of episodic rewards, prior intention STD, posterior intention STD, habitual ratio, number of iterations in AIf, and free-energy loss, respectively. **g-i** Their mean values in stage 2. **m-r** Their mean values in stage 3. The results are from 20 random seeds for each case, and two-sided Welch's $t$-test is used for statistical comparison

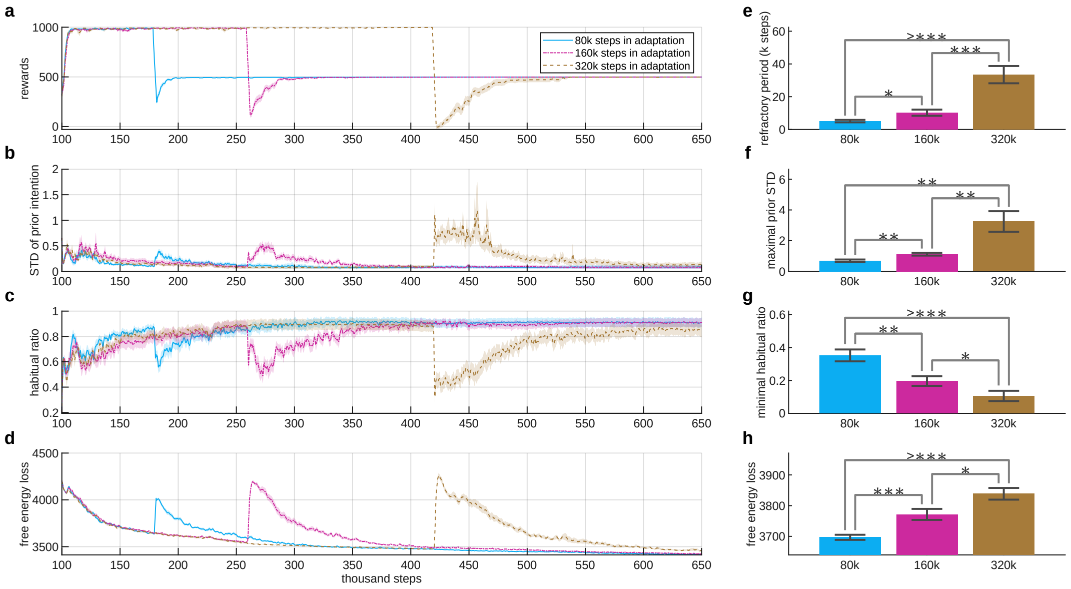


**Supplementary Figure 3.** **Similar results as Figure 3 are observed with small precision threshold $\sigma^{\mathbf{thres}} = 0.01$. a-d** change of rewards, STD of prior intention, habitual ratio and average free energy loss during the adaptation-re-adaptation process, plotted in the same way as in Fig. 2. Curves are smoothed for clarity. **e** Steps (in thousand) needed to change to re-adapt its behavior for new reward configuration (reaching 80% optimal performance). **f** Maximal natural logarithm of STD of the prior intention in re-adaptation stage. **g** Minimal habitual ratio in re-adaptation stage. **h** Average free energy loss in the first 100k steps of re-adaptation stage. Data are presented as mean value ± standard error of the mean, and two-sided Welch's $t$-test is used for statistical comparison (n=20 random seeds for each case).

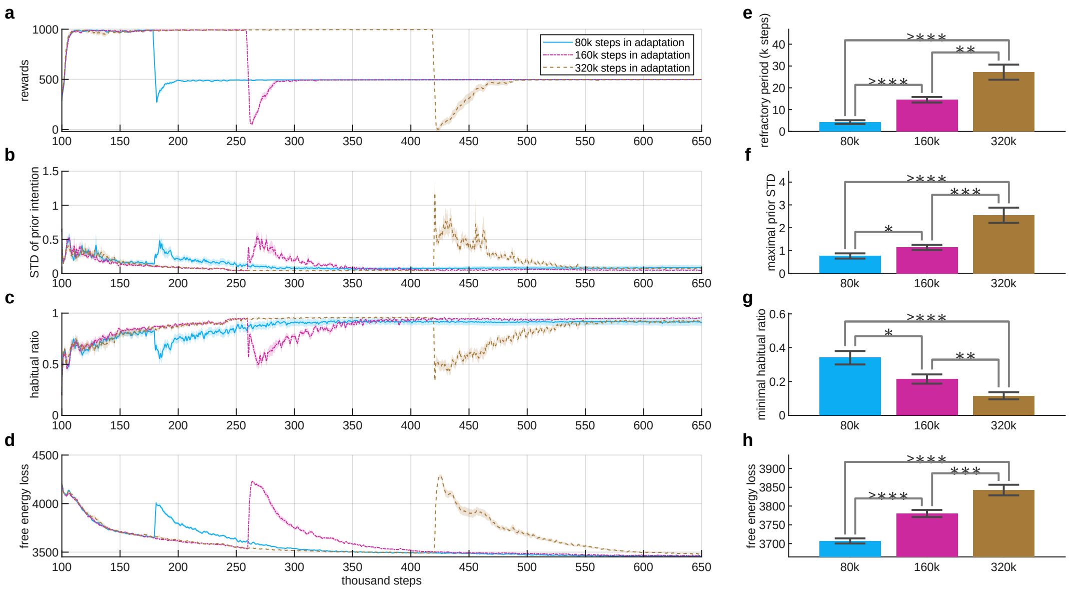


**Supplementary Figure 4. Similar results as Figure 3 are observed with large precision threshold $\sigma^{\mathbf{thres}} = 0.2$. a-d** change of rewards, STD of prior intention, habitual ratio and average free energy loss during the adaptation-re-adaptation process, plotted in the same way as in Fig. 2. Curves are smoothed for clarity. **e** Steps (in thousand) needed to change to re-adapt its behavior for new reward configuration (reaching 80% optimal performance). **f** Maximal natural logarithm of STD of the prior intention in re-adaptation stage. **g** Minimal habitual ratio in re-adaptation stage. **h** Average free energy loss in the first 100k steps of re-adaptation stage. Data are presented as mean value ± standard error of the mean, and two-sided Welch's $t$-test is used for statistical comparison (n=20 random seeds for each case).

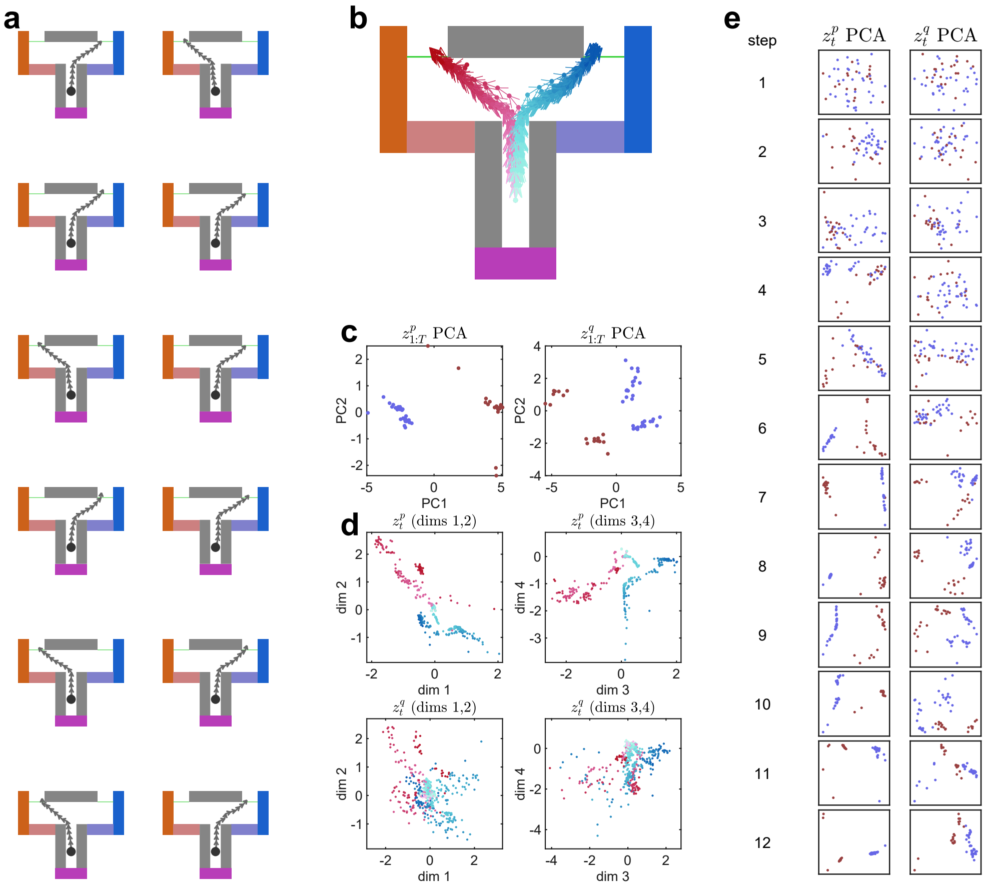


**Supplementary Figure 5. Visualization of the detail behavior and internal representation** learned after the adaptation stage in the second experiment (Figure 4a). **a** Moving trajectories (aerial view) of an example agent's behavior in 12 different trials. The black dot denotes the initial position of the agent and each arrow denotes one step. **b** The example agent's moving trajectories of multiple trials. The color indicates the final escaping exit (red or blue) and step from the start (lightness). **c** Visualizing internal representations of the agent using principal component analysis (PCA) of the flattened intention sequence $z^q_{1:T}$ and $z^p_{1:T}$ in each trial. The first 2 PCs are plotted, where colors indicate final exit (red: left, blue: right). **d** Detailed values of prior and posterior intentions. The colors correspond to the position in the maze, consistent to those shown in panel **b** ($z$ in our study is 4-dimensional). **e** visualization of the first two PCs $z^q_t$, $z^p_t$ in each step from multiple trials. The markers correspond to final exit. All results in this figure are from the same agent model's multiple trials in the end of the adaption stage in the second experiment (Figure 4a).

## Supplementary Reference


1. Higgins, I. *et al.* $\beta$-VAE: Learning basic visual concepts with a constrained variational framework. In *International conference on learning representations* (2017).
2. Huang, Y. & Rao, R. P. Predictive coding. *Wiley Interdiscip. Rev. Cogn. Sci.* **2**, 580–593 (2011).
3. Doya, K. Bifurcations of recurrent neural networks in gradient descent learning. *IEEE Transactions on neural networks* **1**, 218 (1993).
4. Kullback, S. & Leibler, R. A. On information and sufficiency. *The Annals Math. Stat.* **22**, 79–86 (1951).
5. Kingma, D. P. & Welling, M. Auto-encoding variational Bayes. In *Proceedings of the International Conference on Learning Representations (ICLR)* (2014).
6. Friston, K. *et al.* Active inference and learning. *Neurosci. & Biobehav. Rev.* **68**, 862–879 (2016).
7. Friston, K. The free-energy principle: a unified brain theory? *Nat. Rev. Neurosci.* **11**, 127–138 (2010).
8. Daw, N. D., Niv, Y. & Dayan, P. Uncertainty-based competition between prefrontal and dorsolateral striatal systems for behavioral control. *Nat. neuroscience* **8**, 1704–1711 (2005).
9. Schwöbel, S., Marković, D., Smolka, M. N. & Kiebel, S. J. Balancing control: a bayesian interpretation of habitual and goal-directed behavior. *J. mathematical psychology* **100**, 102472 (2021).
10. Schultz, W., Dayan, P. & Montague, P. R. A neural substrate of prediction and reward. *Science* **275**, 1593–1599 (1997).
11. Doya, K. Complementary roles of basal ganglia and cerebellum in learning and motor control. *Curr. opinion neurobiology* **10**, 732–739 (2000).
12. Rangel, A., Camerer, C. & Montague, P. R. A framework for studying the neurobiology of value-based decision making. *Nat. reviews neuroscience* **9**, 545–556 (2008).
13. Lee, D., Seo, H. & Jung, M. W. Neural basis of reinforcement learning and decision making. *Annu. review neuroscience* **35**, 287–308 (2012).
14. Wimmer, G. E. & Shohamy, D. Preference by association: how memory mechanisms in the hippocampus bias decisions. *Science* **338**, 270–273 (2012).
15. Mnih, V. *et al.* Human-level control through deep reinforcement learning. *Nature* **518**, 529 (2015).
16. Friston, K., FitzGerald, T., Rigoli, F., Schwartenbeck, P. & Pezzulo, G. Active inference: a process theory. *Neural Comput.* **29**, 1–49 (2017).
17. Ueltzhöffer, K. Deep active inference. *Biol. Cybern.* **112**, 547–573 (2018).
18. Millidge, B. Deep active inference as variational policy gradients. *J. Math. Psychol.* **96**, 102348 (2020).
19. Fountas, Z., Sajid, N., Mediano, P. A. & Friston, K. Deep active inference agents using Monte-Carlo methods. In *Advances in Neural Information Processing Systems* (2020).
20. Mazzaglia, P., Verbelen, T. & Dhoedt, B. Contrastive active inference. *Adv. Neural Inf. Process. Syst.* **34**, 13870–13882 (2021).
21. Ahmadi, A. & Tani, J. A novel predictive-coding-inspired variational RNN model for online prediction and recognition. *Neural Comput.* 1–50 (2019).
22. QueiSSer, J. F., Jung, M., Matsumoto, T. & Tani, J. Emergence of content-agnostic information processing by a robot using active inference, visual attention, working memory, and planning. *Neural Comput.* **33**, 2353–2407 (2021).
23. Matsumoto, T. & Tani, J. Goal-directed planning for habituated agents by active inference using a variational recurrent neural network. *Entropy* **22**, 564 (2020).
24. Matsumoto, T., Ohata, W., Benureau, F. C. & Tani, J. Goal-directed planning and goal understanding by extended active inference: Evaluation through simulated and physical robot experiments. *Entropy* **24**, 469 (2022).
25. Wirkuttis, N., Ohata, W. & Tani, J. Turn-taking mechanisms in imitative interaction: Robotic social interaction based on the free energy principle. *Entropy* **25**, 263 (2023).
26. Sutton, R. S. *Temporal credit assignment in reinforcement learning*. Ph.D. thesis, University of Massachusetts Amherst (1984).
27. Eppe, M. *et al.* Intelligent problem-solving as integrated hierarchical reinforcement learning. *Nat. Mach. Intell.* 1–10 (2022).

**28.** Bommasani, R. *et al.* On the opportunities and risks of foundation models. *arXiv preprint arXiv:2108.07258* (2021).

**29.** Schmidhuber, J. One big net for everything. *arXiv preprint arXiv:1802.08864* (2018).

**30.** Hochreiter, S. & Schmidhuber, J. Long short-term memory. *Neural Comput.* **9**, 1735–1780 (1997).

**31.** LeCun, Y. A path towards autonomous machine intelligence version 0.9. 2, 2022-06-27. *Open Rev.* **62** (2022).

**32.** Deisenroth, M. & Rasmussen, C. E. Pilco: A model-based and data-efficient approach to policy search. In *Proceedings of the International Conference on Machine Learning*, 465–472 (2011).

**33.** Ha, D. & Schmidhuber, J. Recurrent world models facilitate policy evolution. In Bengio, S. *et al.* (eds.) *Advances in Neural Information Processing Systems 31*, 2450–2462 (Curran Associates, Inc., 2018).

**34.** Kaiser, L. *et al.* Model-based reinforcement learning for atari. *arXiv preprint arXiv:1903.00374* (2019).

**35.** Hafner, D., Lillicrap, T., Ba, J. & Norouzi, M. Dream to control: Learning behaviors by latent imagination. In *International Conference on Learning Representations* (2019).

**36.** Hafner, D. *et al.* Learning latent dynamics for planning from pixels. In *International conference on machine learning*, 2555–2565 (PMLR, 2019).

**37.** Ke, N. R. *et al.* Learning dynamics model in reinforcement learning by incorporating the long term future. *arXiv preprint arXiv:1903.01599* (2019).

**38.** Schrittwieser, J. *et al.* Mastering Atari, Go, chess and Shogi by planning with a learned model. *arXiv preprint arXiv:1911.08265* (2019).

**39.** Igl, M., Zintgraf, L., Le, T. A., Wood, F. & Whiteson, S. Deep variational reinforcement learning for POMDPs. *arXiv preprint arXiv:1806.02426* (2018).

**40.** Han, D., Doya, K. & Tani, J. Variational recurrent models for solving partially observable control tasks. In *International Conference on Learning Representations* (2020).

**41.** Lee, A., Nagabandi, A., Abbeel, P. & Levine, S. Stochastic latent actor-critic: Deep reinforcement learning with a latent variable model. In *Advances in Neural Information Processing Systems*, vol. 33 (2020).

**42.** Friston, K. J., Daunizeau, J., Kilner, J. & Kiebel, S. J. Action and behavior: a free-energy formulation. *Biol. Cybern.* **102**, 227–260 (2010).

**43.** Sohn, K., Lee, H. & Yan, X. Learning structured output representation using deep conditional generative models. *Adv. neural information processing systems* **28** (2015).

**44.** Alemi, A. A., Fischer, I., Dillon, J. V. & Murphy, K. Deep variational information bottleneck. In *International Conference on Learning Representations* (2017).

**45.** Chung, J. *et al.* A recurrent latent variable model for sequential data. In *Advances in Neural Information Processing Systems*, 2980–2988 (2015).

**46.** Han, D. *et al.* Variational oracle guiding for reinforcement learning. In *International Conference on Learning Representations* (2022).

**47.** Liu, M., Zhu, M. & Zhang, W. Goal-conditioned reinforcement learning: Problems and solutions. *arXiv preprint arXiv:2201.08299* (2022).

**48.** Florensa, C., Held, D., Geng, X. & Abbeel, P. Automatic goal generation for reinforcement learning agents. In *International conference on machine learning*, 1515–1528 (PMLR, 2018).

**49.** Andrychowicz, M. *et al.* Hindsight experience replay. In *Advances in Neural Information Processing Systems*, vol. 30 (2017).

**50.** Luketina, J. *et al.* A survey of reinforcement learning informed by natural language. In *Proceedings of the Twenty-Eighth International Joint Conference on Artificial Intelligence, IJCAI 2019, August 10-16 2019, Macao, China.*, vol. 57, 6309–6317 (AAAI Press (Association for the Advancement of Artificial Intelligence), 2019).

**51.** Levine, S. Reinforcement learning and control as probabilistic inference: Tutorial and review (2018).

**52.** Haarnoja, T., Zhou, A., Abbeel, P. & Levine, S. Soft actor-critic: Off-policy maximum entropy deep reinforcement learning with a stochastic actor. In *International Conference on Machine Learning*, 1856–1865 (2018).

**53.** Millidge, B., Tschantz, A., Seth, A. K. & Buckley, C. L. On the relationship between active inference and control as inference. In *International Workshop on Active Inference*, 3–11 (Springer, 2020).

**54.** Hafner, D. *et al.* Action and perception as divergence minimization. *arXiv preprint arXiv:2009.01791* (2020).

**55.** Achiam, J., Edwards, H., Amodei, D. & Abbeel, P. Variational option discovery algorithms. *arXiv preprint arXiv:1807.10299* (2018).

**56.** Gregor, K., Rezende, D. J. & Wierstra, D. Variational intrinsic control. *arXiv preprint arXiv:1611.07507* (2016).

**57.** Eysenbach, B., Gupta, A., Ibarz, J. & Levine, S. Diversity is all you need: Learning skills without a reward function. In *International Conference on Learning Representations* (2019).

**58.** Sharma, A., Gu, S., Levine, S., Kumar, V. & Hausman, K. Dynamics-aware unsupervised discovery of skills. In *International Conference on Learning Representations* (2020).

**59.** Xu, K., Verma, S., Finn, C. & Levine, S. Continual learning of control primitives: Skill discovery via reset-games. In *Advances in Neural Information Processing Systems*, vol. 33 (2020).

**60.** Mendonca, R., Rybkin, O., Daniilidis, K., Hafner, D. & Pathak, D. Discovering and achieving goals via world models. *Adv. Neural Inf. Process. Syst.* **34**, 24379–24391 (2021).

**61.** McCarthy, J. & Hayes, P. J. Some philosophical problems from the standpoint of artificial intelligence. In *Readings in artificial intelligence*, 431–450 (Elsevier, 1981).

**62.** Reinke, C., Etcheverry, M. & Oudeyer, P.-Y. Intrinsically motivated discovery of diverse patterns in self-organizing systems. In *International Conference on Learning Representations* (2020).

**63.** Tishby, N. & Zaslavsky, N. Deep learning and the information bottleneck principle. In *2015 IEEE Information Theory Workshop (ITW)*, 1–5 (IEEE, 2015).

**64.** Rao, R. P. & Ballard, D. H. Predictive coding in the visual cortex: a functional interpretation of some extra-classical receptive-field effects. *Nat. Neurosci.* **2**, 79 (1999).

**65.** Murray, J. D. *et al.* A hierarchy of intrinsic timescales across primate cortex. *Nat. Neurosci.* **17**, 1661 (2014).

**66.** Liu, Z. *et al.* Swin transformer: Hierarchical vision transformer using shifted windows. In *Proceedings of the IEEE/CVF international conference on computer vision*, 10012–10022 (2021).

**67.** Dolan, R. J. & Dayan, P. Goals and habits in the brain. *Neuron* **80**, 312–325 (2013).

**68.** Van de Cruys, S. *et al.* Precise minds in uncertain worlds: predictive coding in autism. *Psychol. review* **121**, 649 (2014).

**69.** Wunderlich, K., Smittenaar, P. & Dolan, R. J. Dopamine enhances model-based over model-free choice behavior. *Neuron* **75**, 418–424 (2012).

**70.** Wunderlich, K., Dayan, P. & Dolan, R. J. Mapping value based planning and extensively trained choice in the human brain. *Nat. neuroscience* **15**, 786–791 (2012).

**71.** Cotzias, G. C., Papavasiliou, P. S. & Gellene, R. Modification of parkinsonism—chronic treatment with L-dopa. *New Engl. J. Medicine* **280**, 337–345 (1969).

**72.** Perlmutter, J. S. & Mink, J. W. Deep brain stimulation. *Annu. Rev. Neurosci.* **29**, 229–257 (2006).

**73.** Azulay, J.-P. *et al.* Visual control of locomotion in parkinson's disease. *Brain* **122**, 111–120 (1999).

**74.** Muñoz-Hellín, E., Cano-de-la Cuerda, R. & Miangolarra-Page, J. C. Visual cues as a therapeutic tool in parkinson's disease. a systematic review. *Revista espanola de geriatria y gerontologia* **48**, 190–197 (2013).

**75.** Pellicano, E. & Burr, D. When the world becomes 'too real': a Bayesian explanation of autistic perception. *Trends cognitive sciences* **16**, 504–510 (2012).

**76.** Wild, K. S., Poliakoff, E., Jerrison, A. & Gowen, E. Goal-directed and goal-less imitation in autism spectrum disorder. *J. autism developmental disorders* **42**, 1739–1749 (2012).

**77.** Palmer, C. J., Lawson, R. P. & Hohwy, J. Bayesian approaches to autism: Towards volatility, action, and behavior. *Psychol. bulletin* **143**, 521 (2017).

**78.** Soda, T. *et al.* Simulating developmental diversity: Impact of neural stochasticity on atypical flexibility and hierarchy. *Front. Psychiatry* **14**, 361 (2023).

**79.** Brown, T. *et al.* Language models are few-shot learners. In *Advances in Neural Information Processing Systems* (2020).

**80.** Radford, A. *et al.* Learning transferable visual models from natural language supervision. In *International conference on machine learning*, 8748–8763 (PMLR, 2021).
